# Supplementary material for: Interleukin-10 contributes to PGE2 signalling through upregulation of EP4 via SHIP1 and STAT3
Source: PLoS One. 2020 Apr 2;15(4):e0230427. doi: 10.1371/journal.pone.0230427 (PMC7117666; doi:10.1371/journal.pone.0230427)
Supplement: S1 Raw images — (PDF) [file pone.0230427.s001.pdf]

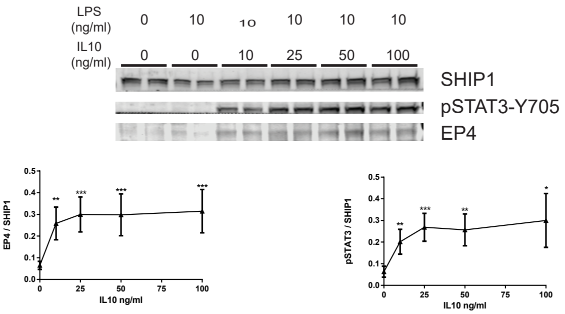

Raw Data

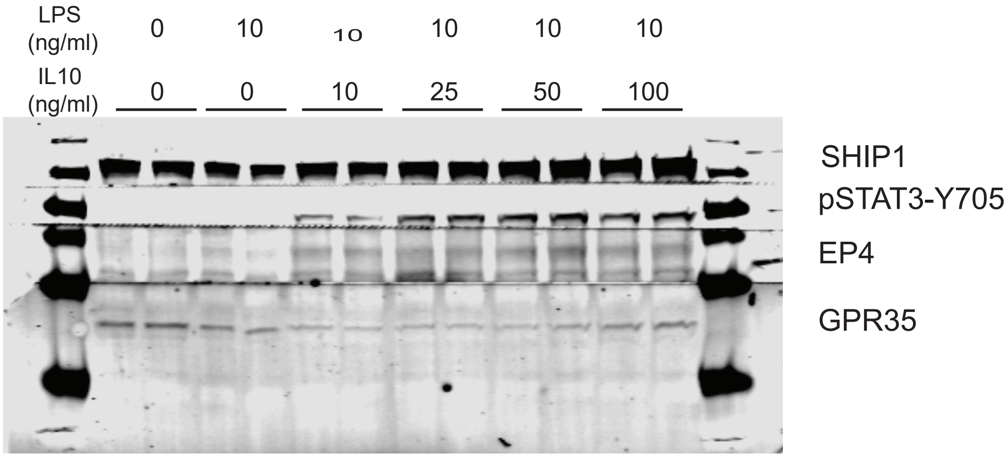

Figure 2

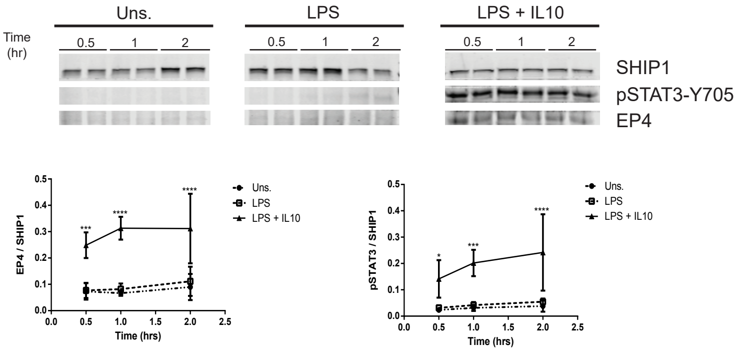

Raw Data

Exposure 1

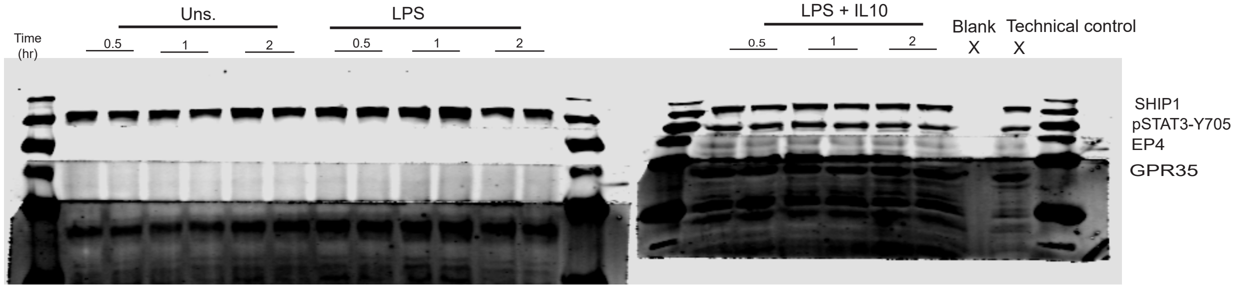

Exposure 2

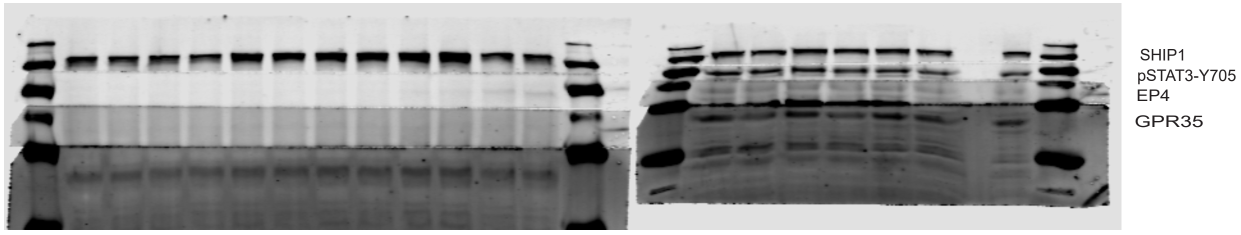

Figure 3

(A) SHIP1 +/+ and -/- perimacs

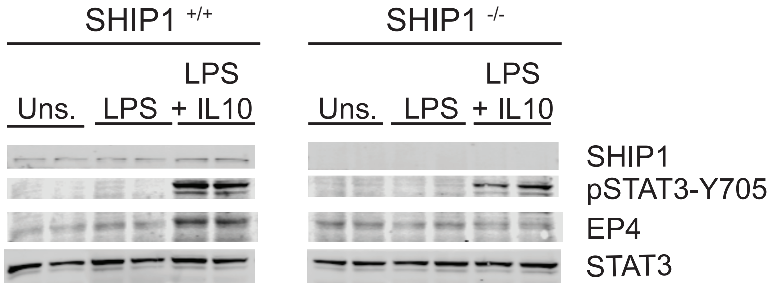

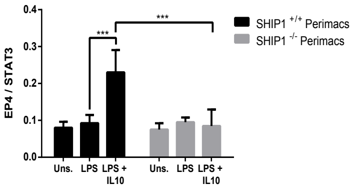

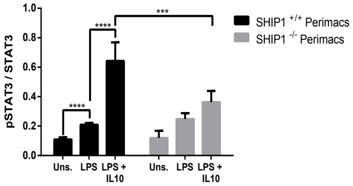

Raw Data

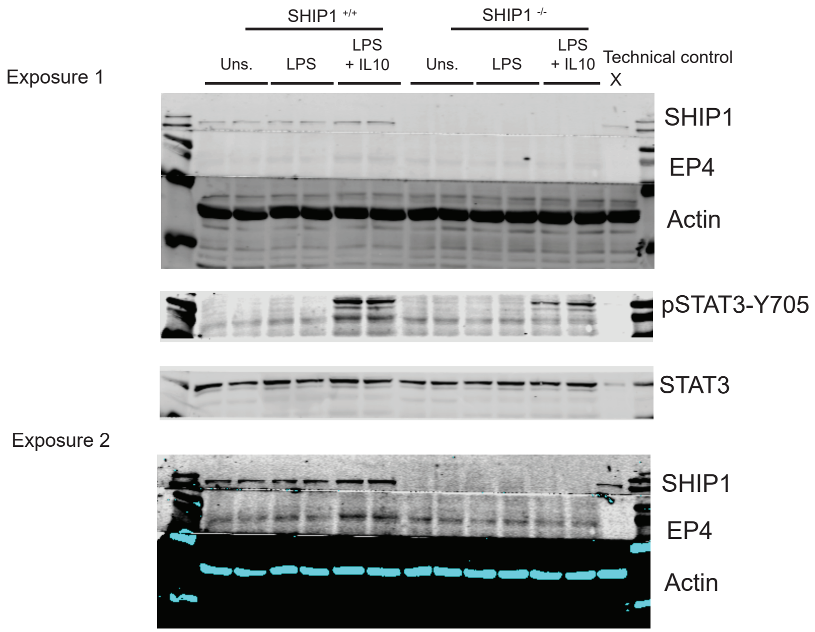

Figure 4A

(B) SHIP1 +/+ and -/- BMDMs

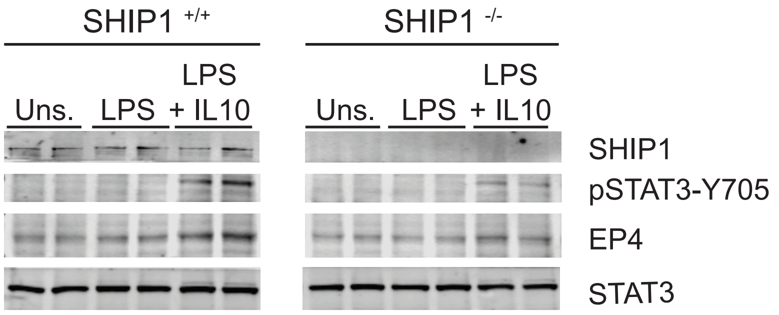

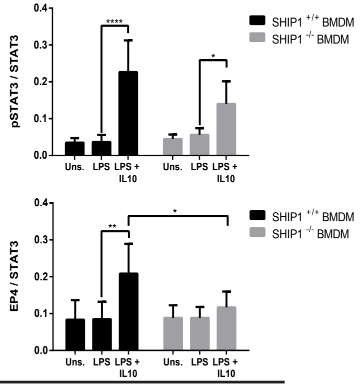

Raw Data

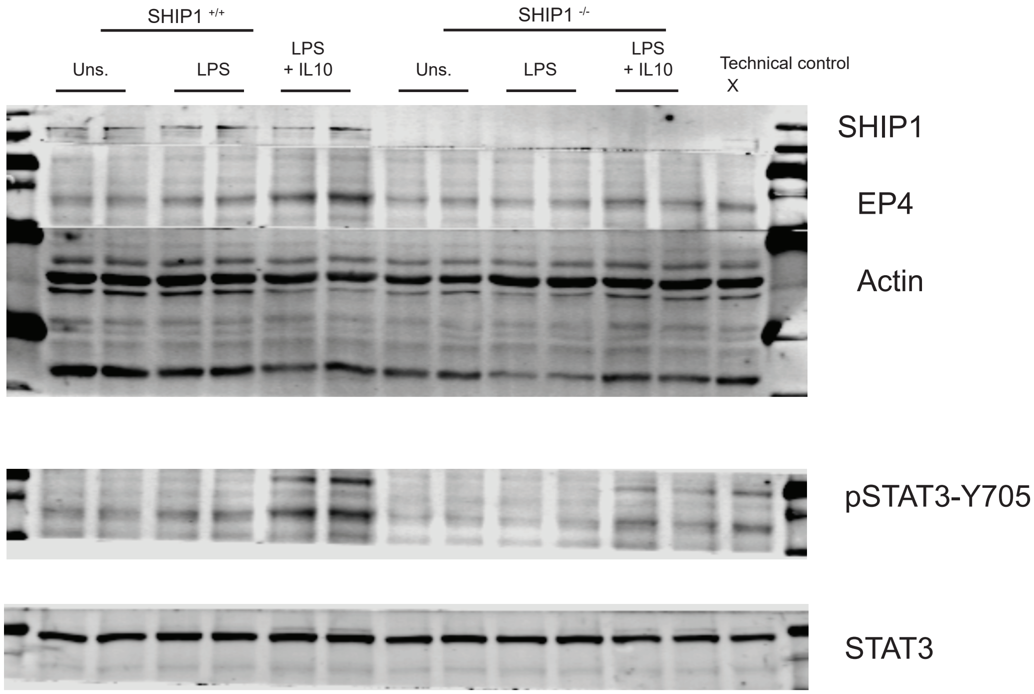

Figure 4B

(C) STAT3 +/+ and -/- BMDMs

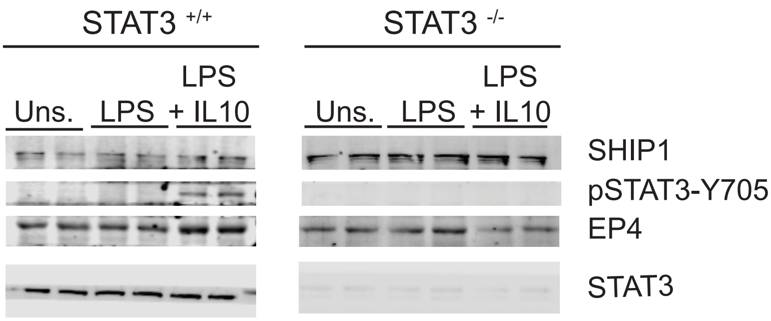

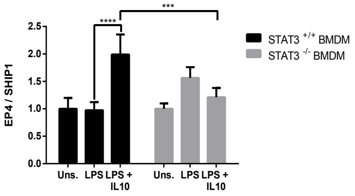

Raw Data

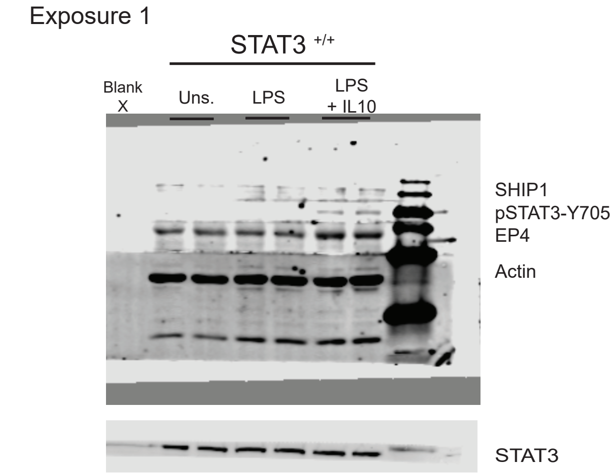

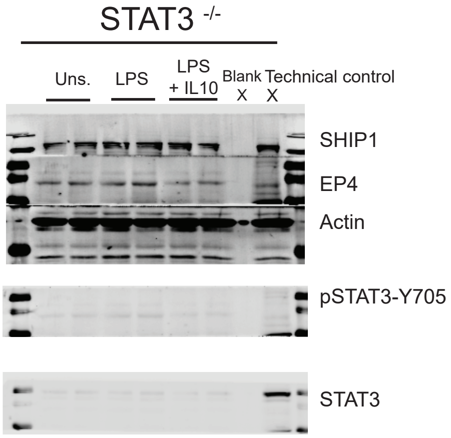

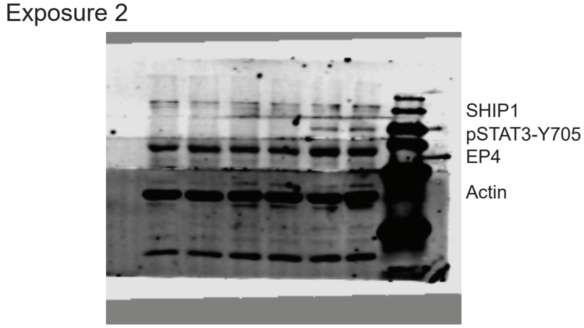

Figure 4C

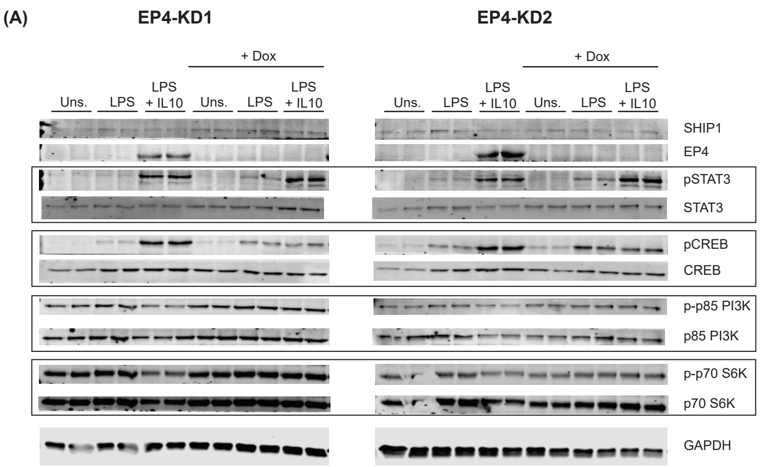

Raw Data

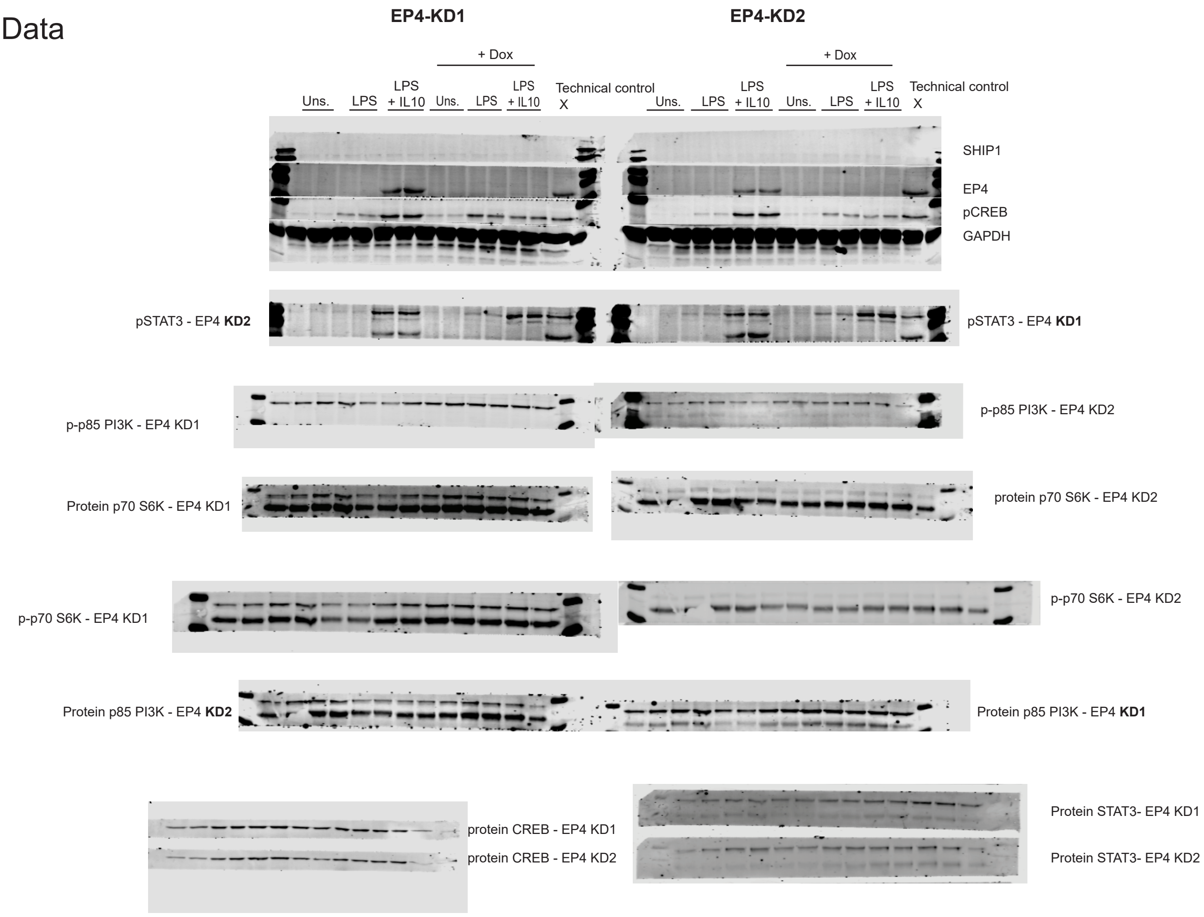

Figure 6A
